# Equity implications of coverage and use of insecticide treated nets distributed for free or with co-payment in two districts in Tanzania: A cross-sectional comparative household survey

**DOI:** 10.1186/1475-9276-10-29

**Published:** 2011-07-21

**Authors:** George M Ruhago, Phares GM Mujinja, Ole F Norheim

**Affiliations:** 1 Department of Health, Health Planning and Research Section, Ilala Municipal, Dar es Salaam, Tanzania; 2 School of Public Health and Social Sciences, Muhimbili University, Dar es Salaam, Tanzania; 3 Division for Medical Ethics, Department of Public Health and Centre for International Health, Kalfarveien 18, University of Bergen, 5018 Bergen, Norway

## Abstract

**Background:**

In Tanzania, the distribution and coverage of insecticide-treated nets (ITNs) is inequitable. Arguments about the most effective and equitable approach to distributing ITNs centre around whether to provide ITNs free of charge or continue with existing social marketing strategies. The Government has decided to provide free ITNs to all children under five in the country. It is still uncertain whether this strategy will achieve equitable coverage and use. This study examined the equity implications of ownership and use of ITNs in households from different socioeconomic quintiles in a district with free ITNs and a district without free ITN distribution.

**Methods:**

A cross-sectional comparative household survey was conducted in two districts: Mpanda in Rukwa Region (with free ITN roll out) and Kisarawe in Coast region (without free ITNs). Heads of 314 households were interviewed in Mpanda and Kisarawe. The concentration index was estimated and regression analysis was performed to compare socioeconomic inequalities in ownership and use of ITNs.

**Results:**

Ownership of ITNs increased from 29% in the 2007/08 national survey to 90% after the roll out of free ITNs in Mpanda, and use increased from 13% to 77%. Inequality was considerably lower in Mpanda, with nearly perfect equality in use (concentration index 0.009) and ownership (concentration index 0.010). In Kisarawe, ownership of ITNs increased from 48% in the 2007/08 national survey to 53%, with a marked inequality concentration index 0.132. ITN use in Kisarawe district was 42% with a pro rich concentration index of 0.027.

**Conclusions:**

The results shed some light on the possibilities of reducing inequality in ownership and use of ITNs and attaining Roll Back Malaria and Millennium Development Goals through the provision of free ITNs to all. This has the potential to decrease the burden of disease and reduce disparity in disease outcome.

## Background

This study concerns the equity implications of different distribution strategies for insecticide treated mosquito nets in Tanzania, with a particular focus on their coverage and use. There are an estimated 247 million malaria cases with 3.3 billion people at risk of malaria worldwide. Over half of the cases are in Africa south of the Sahara and these cause nearly a million deaths of which over 80% are of children under five years of age [1].

Tanzania is one of the countries with the highest burden of malaria, ranked third in the world by WHO on the basis of malaria incidence and mortality rates, after Nigeria and the Democratic Republic of Congo [1]. The disease is endemic in almost all parts of the country, with more than 90% (39 million) of the population in Tanzania at risk of getting malaria [2]. Malaria is a major cause of mortality in children under five years old. In Tanzania, there are more than 100,000 deaths due to malaria per year, about 80% of which are children under five[3]. Malaria also contributes substantially to maternal mortality. The disease is considered to be one of the main causes of poverty in Tanzania and elsewhere in Africa. Malaria consumes about 3.4% of GDP per year in Tanzania, resources that could have been used to meet other equally critical public needs [4].

In a systematic review, it has been found that Insecticide Treated Nets (ITNs) are one of the main tools in combating malaria, along with other interventions such as prompt and effective treatment, use of intermittent preventive treatment of malaria among pregnant women and spraying houses with insecticides. ITNs are estimated to be able to reduce child mortality by 17% and clinical episodes by 50% among users [5]. However, fifteen years after this evidence of ITN use reducing child mortality and morbidity was published, coverage and use of ITNs in Tanzania is still low. In a 2007/08 survey, about 39% of households had at least one ITN, and only 26% of children under five years of age slept under an ITN [6].

The Abuja declaration and Millennium Development Goals require that those at risk of malaria be protected against the disease. The Roll Back Malaria Partnership, the US President's Malaria Initiative (PMI), the Global Fund to Fight AIDS, Tuberculosis and Malaria and the World Bank aim to scale up coverage and use of Insecticide Treated Nets (ITNs) to at least 80% among young children and pregnant women by 2015 [1,7,8].

However, the inequity in coverage and use of proven cost-effective interventions like ITNs may jeopardize the achievement of the Millennium Development Goals and the Roll Back Malaria Targets [9]. Studies show that there is inequity in ITN coverage and use between various socio-economic groups, with the poorest benefiting least from ITNs; even where these are highly subsidized [10]. There is strong evidence to suggest that a household malaria episode is associated with reduced household socioeconomic status [11,12]. Without equity in accessing ITNs, this important public health tool will have little impact on the intended outcomes [13].

Much of the public discussion is focused on designing a distribution system for ITNs that will achieve higher coverage at lower cost and is affordable to the end user, regardless of their socioeconomic status. Should ITNs be distributed free of charge, through social marketing or through commercial markets? Until recently there was general agreement that subsidized access to ITNs should be provided on a large scale and in the long term, but disagreement exists over how these methods of providing subsidies should be deployed [14]. Both free distribution and social marketing have their advantages and disadvantages. While the former is criticized for lack of sustainability after donor withdrawal, social marketing probably has the disadvantage of making nets inaccessible to the poor because of the costs involved.

Most studies of ITN ownership and use do not directly compare coverage and use between delivery systems, so it is difficult to make conclusions about the relative merits of each delivery system [15].

This paper presents a comparison of the socioeconomic inequity in ownership and use of ITNs in an area where they are provided free of charge compared to an area where they are purchased, in Tanzania.

## Methods

### Study area

The study was carried out in two districts purposively chosen to represent the range of ITN delivery mechanisms present in Tanzania. Both Kisarawe and Mpanda districts are malaria endemic with similar socio-demographic characteristics. The study was carried out in March and April 2009 during the rainy season when there is peak malaria transmission. Data collection was conducted six months after households with children under five in Mpanda had received free ITNs from a nationwide campaign distributing free ITNs to all children under five years of age which started in October 2008. Mpanda has few established social marketing and private outlets. During data collection, the national campaign for distributing free ITNs had not yet reached Kisarawe, Kisarawe represents a district without free ITNs but it has a well established social marketing system with national campaigns and some local NGOs (such as Plan International) providing heavily subsidized ITNs. There are also commercial outlets for bed nets. There is a reliable supply because of the area's location close to Tanzania's commercial capital, Dar es Salaam.

### Study design and Population

A quantitative comparative cross sectional household survey was carried out, involving head of households with children under five years or their representative when the head of households were not present at the time of the study. Sample size was calculated on the basis of the estimated proportion of children under five sleeping under Insecticide Treated Nets (ITNs) in Tanzania (26%) [6]. After adjustment for non response, we aimed to interview a sample of 330 households.

A simple random sampling procedure was employed in selecting study participants; one division in each district was randomly selected from a list of all divisions, two wards (one rural and one urban) from each selected division. By using simple random sampling, two villages for rural wards and two streets for urban wards were selected. For each village/street that was randomly selected, all households with children under five years were enumerated and randomly selected. A total of 314 (95.2%) households were interviewed: 158 (96%) in Mpanda and 156 (95%) in Kisarawe. Non response was largely due to the absence of an adult at home. Every head of the selected household either female or male present at home was interviewed. Ethical clearance was sought and acquired from the Muhimbili University of Health and Allied Sciences (MUHAS) research and publication committee. Permission to conduct research was sought from the Regional Administrative Secretaries of both Rukwa and Coast regions. Written consent to participate was sought from all participants.

### Data analysis

All data were cleaned and analysed using STATA Version 10.0 (Stata Corporation, 2007). STATA was used in computing frequencies, calculating confidence intervals, chi-square tests and regression analysis to compare differences in proportions of ITN ownership and use between groups with different socioeconomic status and between study areas. We also used Principal Component Analysis (PCA) to generate the wealth index. A p-value of 0.05 was set for statistical significance.

#### Principal component analysis

The PCA was used to develop wealth indices for the study households based on household characteristics, ownership of assets, source of drinking water, education level and occupation of head of household[16-18]. Household characteristics included ownership of the house, number of sleeping rooms, composition of the floor and type of toilet facility. Household assets included iron, radio, television, telephone, land and bicycle. Variables that featured in only a few households were not included in the principal component analysis, such as car, motorcycle and refrigerator. To allow for an adequate capture of each district's wealth differences, the PCA scores were generated separately for each district. The first principal component accounted for 28.7% variation in Mpanda district, with the first eigenvalue of 3.4. In Kisarawe the first principal component described 26.7% variation with first eigenvalue of 3.2. Households were ranked into five socioeconomic quintiles; most poor, poorer, poor, and less poor and least poor.

#### Equity analysis

Concentration curves and the concentration index as described in O'Donnell, O. et al [18], and logistic regression [19], were used to determine the extent of socio-economic inequality in the ownership and use of mosquito nets. The concentration curve illustrates the degree of socioeconomic inequality in the health variable, and whether it is more prominent in poor or less poor quintiles. The concentration index quantifies the extent of the health inequality, enabling measurement and comparison between the districts [20].

The concentration index ranges from -1 to +1 with a value of 0 indicating absence of socioeconomic related inequality. Masanja et al describe health variables as "Goods" when they are desirable, such as coverage for ITNs, and as "Bads", when they are undesirable, such as malnutrition or mortality [21]. In this study of a good health variable, the index takes a negative value if coverage and use is concentrated among the poorest and a positive value if the variable is concentrated among the least poor group [22].

## Results

A total of 890 and 798 people lived in the visited households in the study areas in Mpanda and Kisarawe districts, respectively. Children under five constituted about 32.6% (290) and 26.4% (211) of all visited household occupants in Mpanda and Kisarawe districts respectively.

### Ownership of mosquito bed net

Mosquito bed net ownership varied significantly between the two districts. A household in Mpanda was more likely to own a mosquito net (98%) than a household in Kisarawe (76%) (OR 11.97, p < 0.001). A similar pattern was seen for ITN ownership; households in Mpanda (90%) were more likely to own at least one ITN than households in Kisarawe (53%) (OR 4.93, p < 0.001) (Table 1).

**Table 1:** Study Households characteristics of ownership and use of mosquito bed nets.

Districts							
**Characteristic**	**Mpanda**		**Kisarawe**				

***1.1 Ownership of ITN and untreated net***	**N**	**%**	**N**	**%**			

**Total number of nets**	**369**	**100**	**207**	**100**			

ITNs	322	87.3	127	61.4			

Untreated net	47	12.7	80	38.6			

**Total number of HH**	**158**	**100**	**156**	**100**			

HH with any net	155	98.1	119	76.3			

HH with ITN	142	89.9	82	52.6			

HH with untreated net	13	8.2	37	23.7			

HH without net	3	1.9	37	23.7			

***1.2 HH use of ITN and untreated net***							

**Total Number of under fives**	**290**	**100**	**211**	**100**			

Slept under ITN	222	76.6	88	41.7			

Slept under untreated net	22	7.6	30	14.2			

Slept under any net (ITN+ untreated)	244	84.1	118	55.9			

**1.3 Probability of ownership and use of bed net in study House Hold**							
**District**	**Mpanda**		**Kisarawe**				

	**N**	**%**	**N**	**%**	**OR**	**P**	**CI**

HH with any net	155	98.1	119	76.3	11.97	<0.001	(4.2 - 34.5)
HH with ITN	142	89.9	82	52.6	4.93	<0.001	(2.5 - 9.8)
Slept under any net	244	84.1	118	55.9	6.25	<0.001	(3.2-12.0)

**Note: **Do not have a net/ITN(=0)						Own a net/ITN (=1)	

### Mosquito bed net ownership by wealth index

Ownership of mosquito bed net varied significantly between socioeconomic quintiles in Kisarawe. The poorest households were less likely to own an ITN compared with the least poor households, with an ownership proportion of 8.7% for the poorest quintile compared to 17.4% for the least poor. There was a pro-rich concentration index of 0.132 (Table 2).

**Table 2:** Proportion variations in ownership of ITNs and untreated nets, in various social economic groups of study households

*Social economic groups*						
	Poorest	Very poor	Poor	Less Poor	Least Poor	Conc. Index
***1. Mpanda (N = 369)Over all***						
**Proportion ownership % (N) [95% CI]**						
ITN (N = 322)	17.9%(66)[14.1-22.2]	17.1%(63)[13.4-21.3]	15.5%(57)[11.9-19.5]	18.7%(69)[14.9-23.10]	18.2%(67)[14.4-22.5]	0.0102
Untreated net (N = 47)	3.0%(11) [1.5-5.3]	2.2%(8) [0.9-4.2]	2.7%(10)[1.3-4.9]	1.6%(6)[0.6-3.5]	3.2%(12)[1.7-5.6]	0.0075
Any net (N = 369)	20.9%(77)[16.8-25]	19.2%(71)[15.3-23.6]	18.2%(67)[14.4-22.5]	20.3%(75)[16.3-24.8]	21.4%(79)[17.3-26.0]	0.0087

***2.Kisarawe***						
**Proportion ownership % (N) [95% CI]**						
ITN(N = 127)	8.7%(18)[5.2-13.4]	9.2%(19)[5.6-14.0]	12.6%(26)[8.4-17.9]	13.5%(28)[9.2-19.0]	17.4%(36)[12.5-23.4]	0.1318
Untreated Net(N = 80)	3.4%(7)[1.4-6.8]	3.4%(7)[1.4-6.8]	6.8%(14)[3.7-11.1]	8.2%(17)[4.9-12.8]	17.0%(36)[12.1-22.7]	0.303
Any net(=207)	12.1%[25)[8.0-17.3]	12.6%(26)[8.4-17.9]	19.3%(40)[14.2-25.4]	21.7%(45)[16.3-28.0]	34.3%(71)[27.9-41.2]	0.2033

In Mpanda district, there was no significant difference in net ownership between socioeconomic quintiles. Ownership in the poorest quintile was 17.9% compared with 18.2 in the least poor quintile. The concentration index was 0.0102.

**Figure 1:**
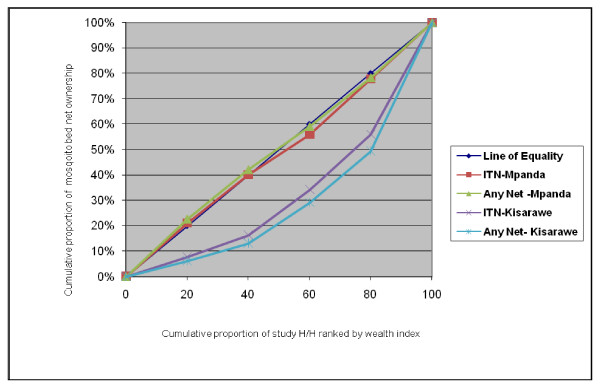
**Degree of inequality for mosquito bednets in household of different socioeconomic status in Mpanda and Kisarawe district**.

The disproportionate distribution of mosquito bed nets is clearly illustrated in the concentration curve (Figure 1).

In Figure 1 the curve far below the line of equality represents ownership of mosquito bed nets (ITNs and any net) in Kisarawe, and it is evident that mosquito bed nets are more concentrated in households in the least poor socioeconomic quintile; so there is a pro-rich inequality. The curve for ITNs and any net in Mpanda lies almost on the line of equality. This indicates nearly perfect equality in mosquito net ownership in that district.

### Mosquito net use

Children under five in Mpanda were more likely to sleep under an ITN (77%) compared to children in Kisarawe district (42%). Use of any net by children under five in Mpanda was higher (84%) compared to (56%) in Kisarawe (OR 6.25, p < 0.001) (Table 1).

### Use of mosquito bed net across socioeconomic groups

Use of mosquito nets varied in Kisarawe. Children under five in household in poorest socioeconomic quintile (6.6%) were less likely to sleep under an ITN than in households of the least poor quintile (9.5%). There was some pro-rich inequality in the use of ITNs among children under five in different socioeconomic quintiles in Kisarawe with a concentration index of 0.027 compared to nearly perfect equality in Mpanda and a concentration index of 0.009 (Table 3). For any net, the difference was almost double; with only (43%) of children under five from the poorest socioeconomic quintile in Kisarawe sleeping under an ITN compared to 78% from the least poor quintile. In Mpanda district, ITN use did not vary significantly between different socioeconomic groups (p = 0.468) (Table 1).

**Table 3:** Proportion variations in use of ITNs and untreated nets, by children under give across different social economic groups in Mpanda and Kisarawe districts

Social economic groups						
	Poorest	Very poor	Poor	Less Poor	Least poor	Conc. index
**1.Mpanda (N = 290)Over all**						

**Proportion use %(N)[95%CI]**						

ITN (N = 222)	15.2%(44)[11.4-19.7]	14.8%(43)[11.1-19.3]	15.5%(45)[11.7-20.0]	16.2(47)[12.3-20.8]	14.8%(43)[11.1-19.3]	0.009
Untreated Net(N = 22)	1.8%/(5)[0.6-3.8]	0.7%(2)[0.11-2.26]	2.1%(6)[3.6-19.6]	1% (3)[0.3-2.8]	2.1%(6)[0.8-4.3]	0.064
Any Net(N = 244)	16.9%(49)[12.9-21.5]	15.5%(45)[11.7-20.0]	17.6%(51)[13.5-22.3]	17.2%(52)[13.2-21.9]	16.9%(48)[12.9-21.5]	0.014

**2.Kisarawe (N = 211) Overall**						

**Proportion use %(N)[95%CI]**						

ITN (N = 88)	6.6%(14)[3.8-10.6]	9%(19)[5.7-13.5]	8.1%(17)[4.9-12.3]	8.5%(18)[5.3-12.9]	9.5%(20)[6.1-14.0]	0.027
Untreated Net(N = 32)	1.4%(3)[0.4-3.8]	1.9%(4)[0.6-4.5]	2.8%(6)[1.2-5.8]	2.4%(5)[0.9-5.2]	5.7%(12)[3.1-9.5]	0.232
Any Net(=118)	8%(17)[4.9-12.3]	10.9%(23)[7.2-15.7]	10.9%(23)[7.2-15.7]	10.9%(23)[7.2-15.7]	15.2%(32)[10.8-20.5]	0.082

**Figure 2:**
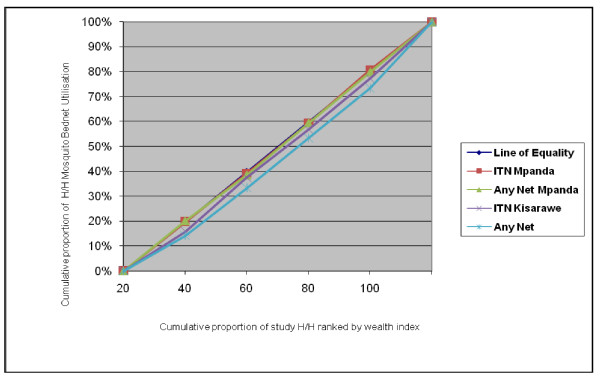
**Degree of Inequality for Mosquito bed net use in H/H of different Socioeconomic Status in Mpanda and Kisarawe District**.

The concentration curve in Figure 2 depicts the variation of mosquito bed net use between different socioeconomic quintiles. Lines of use of ITNs and any net for Kisarawe district lie below the line of equality indicating that mosquito bed net use is pro rich in that district. While for Mpanda district the line for ITN and any net lies on or above the line of equality indicating almost perfect equality in use of ITNs and any net. The difference between the curves is smaller for use than for ownership.

### Source of nets

The major source of bed nets in households from the poorest socioeconomic quintile in Mpanda district was free nets (66%), with only 27% of the households obtaining them from commercial outlets. In households from the least poor socioeconomic quintile, the main sources of nets were from commercial outlets (51%) and from the free distribution system (47%).

Free distribution in Mpanda was pro-poor with a concentration index of -0.064 compared to commercial market sources which was pro-rich with a concentration index of 0.156 (Table 4).

**Table 4:** Source of mosquito ITNs in various study households inMpanda and Kisarawe district by household economic status group

*Source of Net*	*Socio economic groups*					
	**Poorest**	**Very poor**	**Poor**	**Less Poor**	**Least Poor**	**Conc. Index**

**1. Mpanda** **(N = 369) overall**	**(N = 77)**	**(N = 71)**	**(N = 67)**	**(N = 75)**	**(N = 79)**	

**Proportion %(N)[95% CI]**						
Free Nets	66.2%(51)[54.6-76.6]	78.9%56)[67.6-87.7]	61.2%(41)[48.5-72.9]	61.3%(46)[49.4-72.9]	46.8%(37)[35.5-58.4]	-0.064
Commercial	27.3%(21)[17.7-38.6]	19.7%(14)[11.2-30.9]	31.3%(21) [20.6-43.8]	25.3%(19)[16.0-36.7]	50.6%(40)[39.1-62.1]	0.156
Social Market	2.6%(2)[0.3-9.1]	0.00	4.5%(3) [0.9-12.5]	2.7%(2)[0.3-9.3]	2.5%(2)[0.3-8.8]	0.172
Other Source	3.9%(3)[0.8-11.0]	1.4%(1) [0.04-7.6]	2.9% (2) [0.4-10.4]	10.7%(8)[4.7-19.9]	0.00	0.04

**2.Kisarawe** **(N = 207))overall**	**(N = 25)**	**(N = 26)**	**(N = 40)**	**(N = 45)**	**(N = 71)**	

**Proportion %(N)[95% CI]**						
Free Nets	N/A	N/A	N/A	N/A	N/A	N/A
Commercial	16.0%(4)[4.5-36.1]	42.3%(11)[23.4-63.1]	50.0%(20)[33.8-66.2]	51.1%(23)[35.8-66.3]	50.7%(36)[38.6-62.8]	0.269
Social Market	68.0%(17)[46.5-85.1]	46.2%(12)[26.6-66.6]	45.0%(18)[29.0-61.5]	28.9%(13)[16.4-44.3]	36.6%(26)[25.5-49.9]	0.003
Other Source	16.0%(4)[4.5-36.1]	11.5%(3)[2.4-30.2]	5.0%(2)[4.2-26.8]	20.0% (9)[8.0-32.1]	12.7%(9)[6.0-22.7]	0.179

In Kisarawe district; the poorest quintile households mainly depended on getting nets from social marketing (68%) compared to 16% from commercial outlets. For households in the least poor quintile, the main sources of ITNs were commercial (51%) and 37% from social marketing. Social marketing achieved a concentration index of 0.003, while commercial sources exhibited marked inequality with a concentration index of 0.269 (Table 4).

## Discussion

This study shows that the poorest households are less likely to own and use a mosquito bed net. It also shows that free distribution of ITNs is associated with less pro-rich inequality in ownership. Other studies conducted recently in Tanzania also confirm the existence of socioeconomic inequality in ownership and use of ITNs [23-25].

### Mosquito bed net ownership and use after free distribution

The national survey in 2007/08 [6] indicated that ownership of ITNs in Mpanda was 29%. After free distribution, the ownership increased to 90% by the end of 2008; and use of ITNs increased from 13% to 77%. That is beyond the target of 60% coverage set by the Roll Back Malaria African Summit held in Abuja in 2000 [26], and only 3% less than the improved Roll Back Malaria target of 80% coverage by the year 2010 [7] and 2015 by MDG [3]. In Kisarawe District (an area without free ITN distribution); ITN ownership was (53%) almost the same as in the 2007/08 national survey (48%) and far below the MDG and Roll Back Malaria targets.

Ownership and use of ITNs in Mpanda District after free distribution are higher than the levels other studies have found from other settings after free of charge ITN distribution in Kenya, Ghana and Eritrea. In Kenya, ownership increased from 7.1% in 2004 to 67% in 2006 [22]; in Ghana possession was at 74% and use was at 60% [27], and Eritrea possession rose to 82% and use at 68% [28]. However, there are differences between the studies in the length of time between free ITN distribution and the time the study was carried out; two years in Kenya, 38 months in Ghana and one year in Eritrea. Our study was carried out six months after the roll out of free ITNs. Further studies may be required to determine the effect of time on ITN ownership and use after roll-out.

### Mosquito bed net ownership and use by wealth index

The results of this study have indicated that distributing free ITNs enabled households from the poorest socioeconomic quintile to access this important public health intervention. Through increased ownership and use of ITNs, inequalities have been minimized, although more for ownership than for use. In Mpanda, after free ITN distribution, there was nearly perfect equality for ownership. A study in Kenya revealed similar findings where, after distribution of free ITNs, socioeconomic inequity of ownership decreased [22]. In Kisarawe district, ITN use was 40% during the 2007 National survey and remained the same at 42% during this study. However, we cannot, from this preliminary study, conclude that there is causality between free bed net distribution and increased ownership and use of ITNs, but the results shed some light on the significance of providing free ITNs.

### Equity and efficiency of free bed net distribution

Decisions about health service distribution in countries like Tanzania are about how to make sure that interventions achieve equity and efficiency at the same time. In the case of ITNs, the intervention is very cost effective with costs per Disability Adjusted Life Years (DALY) averted ranging between US$ 19 and $ 85 [29]. ITNs in combination with other interventions such as integrated management of childhood illness, vitamin A supplementation, immunisation and exclusive breastfeeding have been associated with reduction in child mortality in Tanzania [30] and can contribute to equity by reducing malaria morbidity and mortality in the poorest quintile households. That highlights the need for increased efforts in distribution of ITNs with explicit pro-poor strategies. Controlling malaria is very costly: resources spent on controlling mosquito bites and preventing malaria by both state and households have been estimated to be US$ 119 million (3.4% of the GDP) in Tanzania [4]. This has also been estimated by the Tanzania National Malaria Control Programme (NMCP). A major share of that expenditure is spent by poor households. The saving could be spent elsewhere to combat poverty and decrease inequality between relatively rich households and the poorest households.

ITNs offer the same public health advantages as vaccines: they are effective, cost-effective and address a large disease burden. It is high time that they received the same approach to public financing [31]. Sustainability can be achieved by combining social marketing with targeting the poorest household with free ITNs Given that many people in African communities share beds and are expected to share freely distributed ITNs, it may prove an effective option in combating malaria [32]. Social marketing through a voucher scheme may remain an important complementary strategy. However, if the equity achieved by free nets is to be sustained, vouchers should be cheaper and more affordable, and with a specific and deliberate strategy of targeting the poorest of the poor. A voucher policy should take into consideration geographical location instead of having a uniform voucher for the whole country. The majority of poor people live in rural areas. Geographical targeting with higher subsidies in rural areas could therefore be considered [31]. Education about the effectiveness of ITN use would create demand and ensure sustainability of ITN coverage and use. It is widespread use of ITNS that will reduce malaria case numbers and ease the demand for health care services.

### Study limitations

This study presents results from a cross sectional study. The main limitation of the study is that it is not a randomized controlled trial and therefore causation cannot be established. The study was conducted just six months after the roll out of free ITNs, so the increased coverage and use may be transient, and there is likelihood that it may drop with time as ITNs wear out. There may be a need for further studies with longer follow up periods to ascertain the sustainability of current coverage rates. However, the strength of this study is that respondents were selected randomly, and the study areas were sufficiently different in ITN delivery mechanisms to allow relevant comparisons. The two districts have similar demographic characteristics and are both Malaria endemic. Ranking of households into different socioeconomic quintiles, however, poses a major challenge. The choice of assets to be included in the construction of a wealth index is difficult as there is no agreed amount or type of asset to be used [18]. Some assets (for example a telephone) may vary considerably in price and may lead to people being placed in the same quintile while their phones are not of the same value. A bicycle, despite being an asset in rural areas, is associated with poverty in urban areas [17,23]. However, according to Filmer and Pritchett [16] a household classified in the poorest group by wealth index will not be classified as rich by a reduction of the number of assets used for classifications of socioeconomic group.

To enhance the reliability of the results, concentration index analysis was supplemented with regression analysis which also revealed inequality in ownership and use of ITNs between different socioeconomic quintiles in an area without free ITN provision.

## Conclusions

The findings of this study indicate that in the areas studied, where ITNs are commercially or socially marketed, there is a low level of ITN ownership and use. In Kisarawe district, ownership is 53% and use 42%. Households of the poorest socioeconomic quintile are the most deprived, with a pro-rich inequality. The study also indicates that free distribution of ITNs is associated with higher rates of ownership and use, and less inequality. A combination of social marketing and distribution of free ITNs to all the poorest households, and deliberate efforts to sustain higher coverage by empowering local capacity building to deliver subsidized ITNs is likely to achieve the goal of combating malaria regardless of socioeconomic status.

### Recommendations for further research

This study provides some information on how the national and international target of 80% coverage and use of ITNs might be achieved, as well as reducing inequality through free distribution of ITNs. Further research should clarify the possible long-term impact of changing distribution policies from cost-sharing schemes to free distribution. The optimal study design would be a controlled randomized trial with a sufficient number of households and adequate time to follow up.

## Competing interests

The authors declare that they have no competing interests.

## Authors' contributions

GMR: Designed the study, collected data, analyzed data and prepared first draft of the manuscript. Some of data presented here form part of GMR's master's thesis.

PGM: Contributed to the design of the study and writing up.

OFM: Contributed to designing, coordinated data analysis and writing up of the manuscript. All authors read and approved the final manuscript.
